# Case report: Pheochromocytoma complicated by type B aortic dissection

**DOI:** 10.3389/fcvm.2023.1236896

**Published:** 2023-09-27

**Authors:** Dan Yi, Xiatian Liu, Libin Fan

**Affiliations:** ^1^ Department of Ultrasound, Shaoxing People’s Hospital, Shaoxing, China; ^2^ Department of Vascular Hernia Surgery, Affiliated Hospital of Shaoxing University, Shaoxing, China

**Keywords:** pheochromocytoma, type B, aortic dissection, case report, computed tomography

## Abstract

**Introduction:**

Pheochromocytomas combined with aortic dissections are rare. Treatment of aortic dissection can be complicated by the presence of pheochromocytomas.

**Case presentation:**

we present the case of a 48-year-old male who visited the hospital with chest and back pain for 13 h. Enhanced computed tomography (CT) revealed a type B aortic dissection combined with a left adrenal mass (72 mm). Elevated 24-h urinary vanillylmandelic acid levels can aid in the diagnosis of pheochromocytomas. Aortic dissection due to unstable hypertension secondary to pheochromocytoma is rare and complicates the procedure. Thoracic endovascular aortic repair was performed, and antihypertensive treatments were administered after surgery. After hypertension was addressed and the patient was stable, laparoscopic resection of the adrenal mass was performed.

**Conclusions:**

despite its rarity, it is important to consider pheochromocytoma as a differential factor for unstable hypertension when an aortic dissection is found.

## Introduction

Aortic dissection is a life-threatening condition caused by a tear in the intima, where blood flow splits the intima and creates a false lumen in the aortic wall. Disruption of the normal blood flow may lead to poor organ perfusion, aortic dissection, and advanced aneurysm formation. Risk factors for aortic dissection include smoking, poorly controlled hypertension, male sex, age, and connective tissue disease (1, 2).

Type B aortic dissection (TBAD) occurs distal to the left subclavian artery (LSCA). The early prognosis for TBAD is good, with a 1-year survival rate of 65% for patients. However, the late prognosis is worse because of anatomy-related complications, with a 5-year survival rate of 50% (3). Thoracic endovascular aortic repair (TEVAR) is recommended as the first-line treatment in patients with acute complicated TBAD (4, 5).

Treatment of aortic dissection can be complicated by the presence of pheochromocytomas. Pheochromocytomas are tumors in which the adrenal medulla and other chromophobic tissues of the adrenergic system produce excessive amounts of catecholamines, which can lead to uncontrolled blood pressure (6). Here, we present a case of aortic dissection as the initial presentation of an undiagnosed pheochromocytoma.

## Case presentation

A 48-year-old male patient presented to our clinic with chest and back pain that had persisted for 13 h. The patient complained of sudden onset of pain in the precordial region more than 13 h prior during the course of a meal, which was persistent and dramatic, and then radiated to the abdomen and waist, with profuse sweating, posterior abdominal distension, vomiting of approximately 200 ml of dark brown fluid, no clots, no syncope, no diarrhea, no cough, or expectoration. The etiology of this condition remains unclear. The patient denied any history of hypertension, diabetes mellitus, tumor, trauma, or previous surgery, and had a 30-year history of smoking.

**Figure 1:**
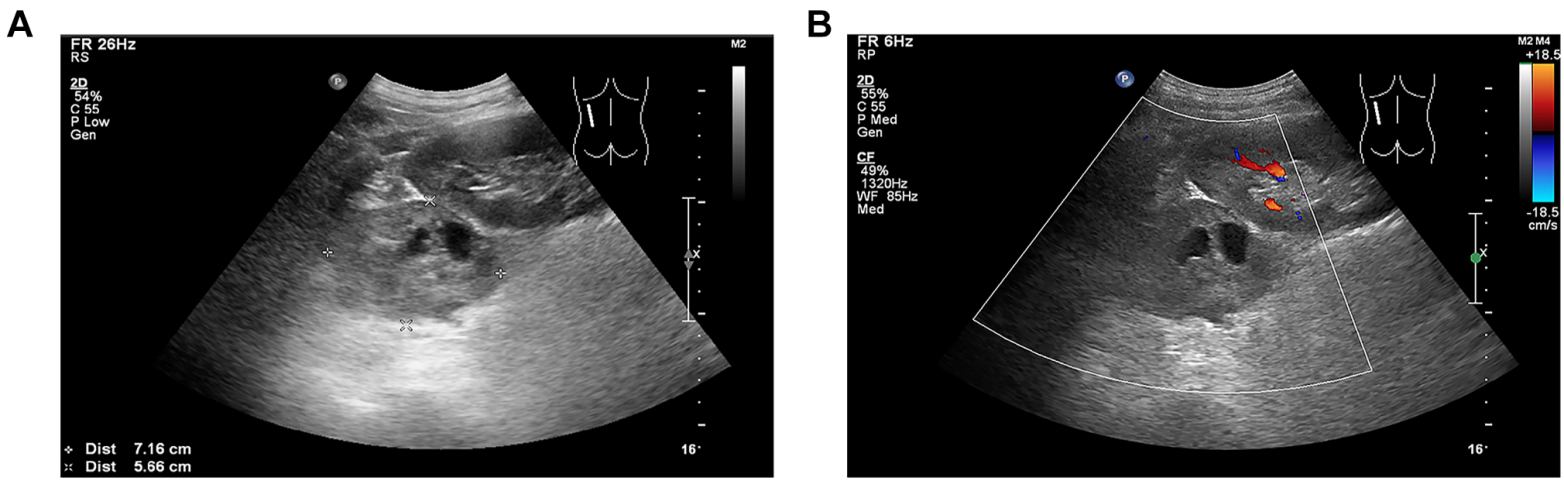
(**A**) Heterogeneous echogenic mass with a size of approximately 72 × 57 mm in the left adrenal gland with clear boundary and regular shape, in which patchy anechoic areas were observed. (**B**) No significant blood flow signal inside the left adrenal mass.

**Figure 2:**
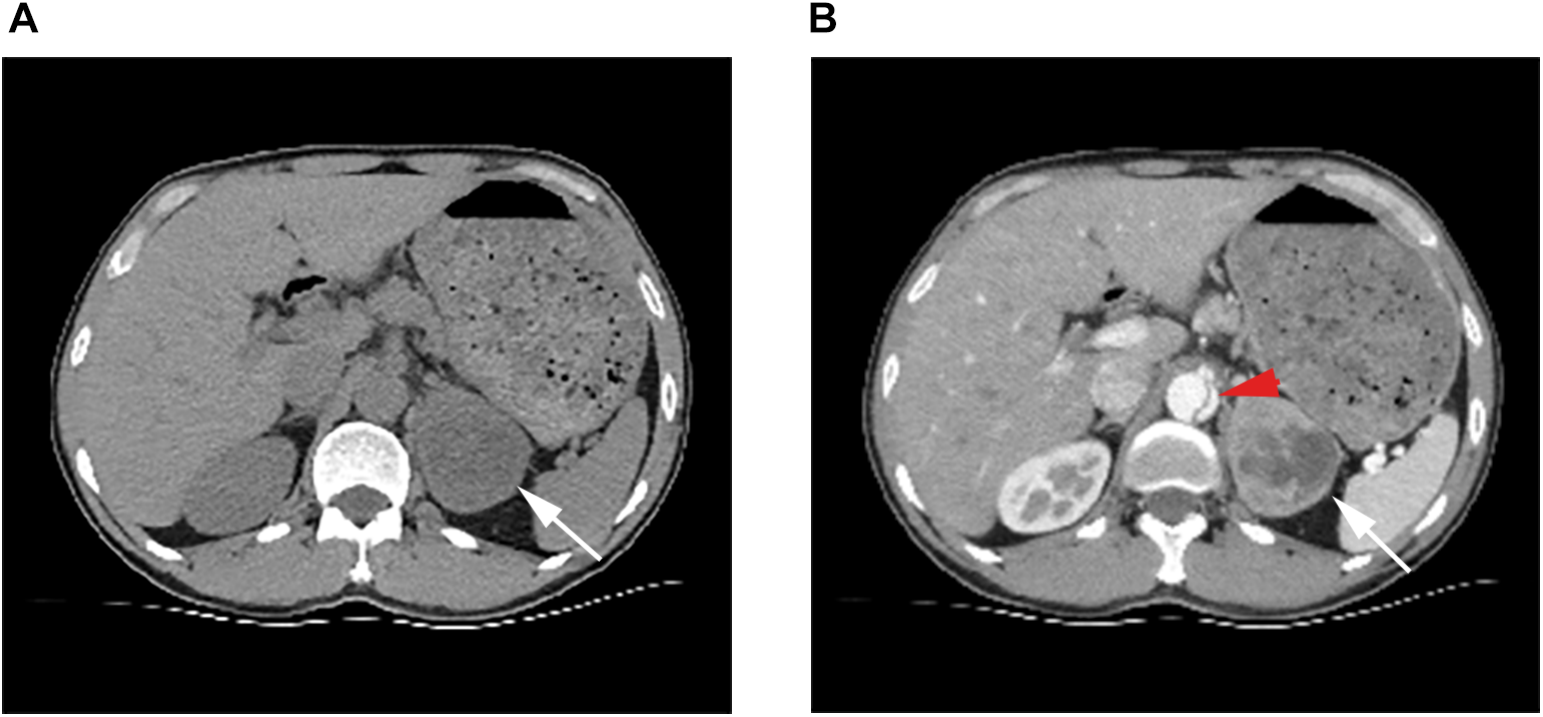
(**A**) Ct scan showing a soft tissue mass in the left adrenal gland with uneven internal density, clear borders, and regular shape. (**B**) White arrows point to the left adrenal mass in the arterial phase, which is significantly enhanced and necrotic inside. Red arrows point to dissection of the abdominal aorta.

**Figure 3:**
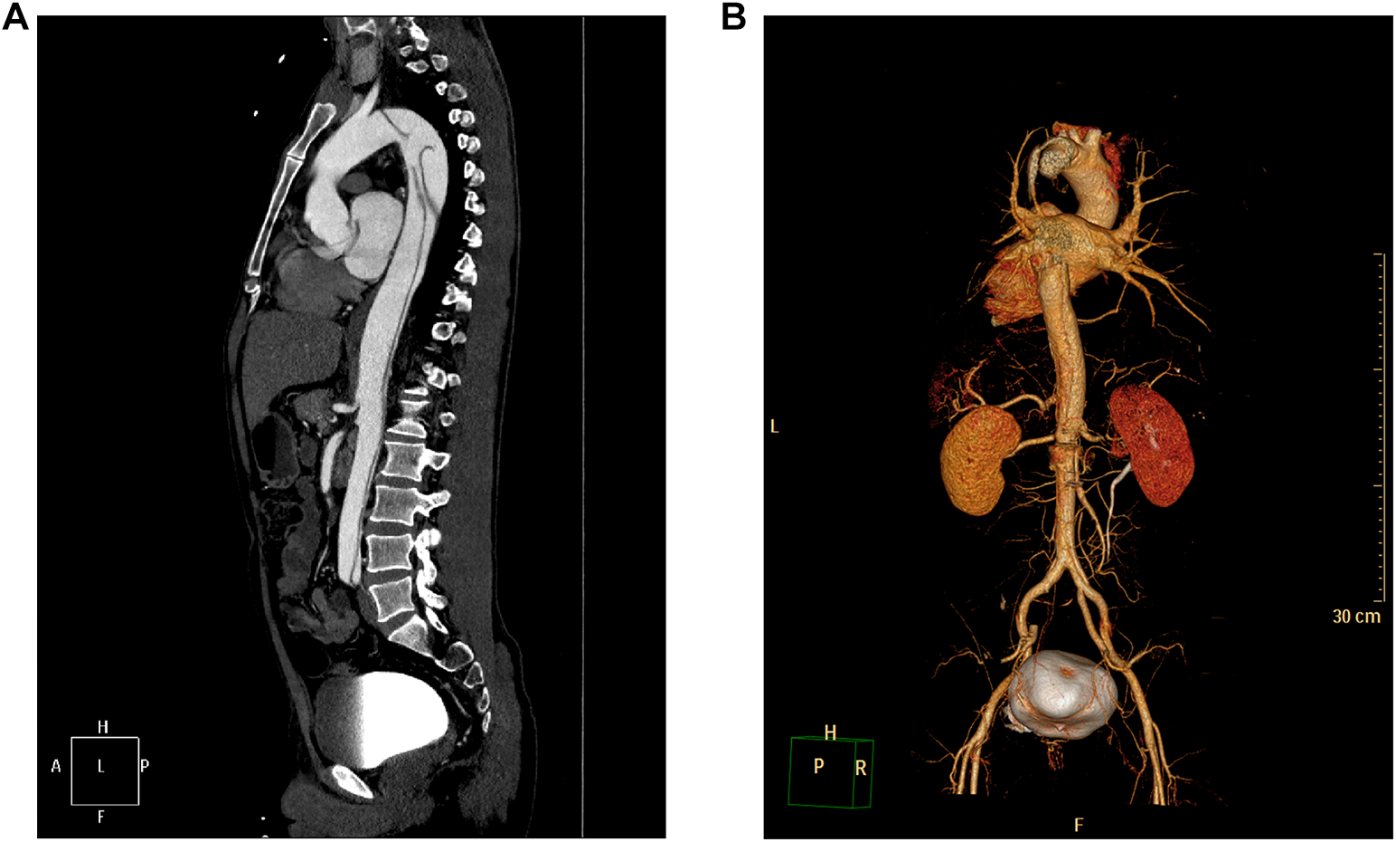
CTA showing medial migration of the intimal sheet from the descending thoracic aorta to the level of both common iliac arteries, and the lumen showing “double-lumen” changes, consistent with aortic dissecting aneurysms (Stanford type B). (**A**) Maximal intensity projection of the sagittal section of the aorta. (**B**) Three-dimensional reconstruction of the aorta.

Physical examination revealed an acutely ill face, clear consciousness, poor spirit, heart rate of 125 beats/min, blood pressure of 210/94 mmHg, distended abdomen, drum sounds on percussion, no tenderness or rebound tenderness, and the presence of femoral and dorsalis pedis pulses. According to the aortic dissection detection risk scoring (ADD-RS) system proposed by the aortic diagnosis and treatment guidelines in 2014, ADD-RS = 1. The etiology of the patient was unknown; clinicians could not rule out aortic dissection, urinary calculi, or other related diseases; therefore, echocardiography and abdominal urinary ultrasonography were performed on the patient. Echocardiography showed normal findings, and abdominal ultrasonography revealed a mass in the left adrenal region (72 × 57 mm, Figure 1). Contrast-enhanced computerized tomography (CT) of the abdomen revealed that the intimal flap moved inward from the descending part of the thoracic aorta to the level of the bilateral common iliac arteries, the lumen showed “double-lumen” changes, the right renal artery opened into the false lumen, and the left renal artery opened into the true lumen, consistent with aortic dissecting aneurysm (Stanford type B). A soft tissue mass was observed in the left adrenal gland, with uneven density, approximately 61 × 53 mm in size, with a clear boundary, and with significant enhancement after contrast-enhanced scanning in which there was necrosis; pheochromocytoma was first considered (Figure 2). The patient subsequently underwent aortic computed tomographic angiography (CTA), which revealed Stanford TBAD (Figure 3). Laboratory tests showed a 24-h urine vanillylmandelic acid (VMA) level of 20.51 mg/24 h (reference value: ≤12 mg/24 h) and a D-dimer level of 6.75 mg/L (reference range 0.0–0.5 mg/L).

Nitroglycerin and esmolol hydrochloride were administered to the patient for antihypertensive treatment after admission and was later replaced with a phentolamine drip due to persistent blood pressure fluctuation. Forty-eight hours after the onset of symptoms, blood pressure control was relatively stable after treatment, and because the patient developed abdominal pain and distension, vomiting of dark brown fluid, and the occult blood test was positive (+ + +) in vomitus, the clinician was concerned about aortic dissection involving visceral malperfusion. After communicating with the patient and his family, the patient actively requested surgery; therefore, thoracic endovascular aortic repair was performed.

Due to concern regarding adrenergic storm caused by intraoperative pheochromocytoma secreting a large amount of catecholamines, a phentolamine drip was given for active antihypertensive treatment before surgery, metoprolol was given to control heart rate, and preoperative blood transfusion preparation was actively performed. The phentolamine drip was continued during the operation and was closely observed by the anesthesiologist, without signs of hemodynamic instability. During the procedure, we sealed the dissection of the descending aorta, with good stent curvature. Multiple digital subtraction angiography (DSA) at the distal end of the stent revealed difficulty in opening the true lumen at the distal end of the graft. The patient's family was informed, and partial artificial blood vessel replacement of the abdominal aorta was recommended. Because of the high surgical risk, the patient's family refused, and requested conservative treatment. Nitroglycerine and esmolol were continued as antihypertensive treatments after surgery, and the blood pressure was stable at 101–154/51–80 mmHg.

**Figure 4:**
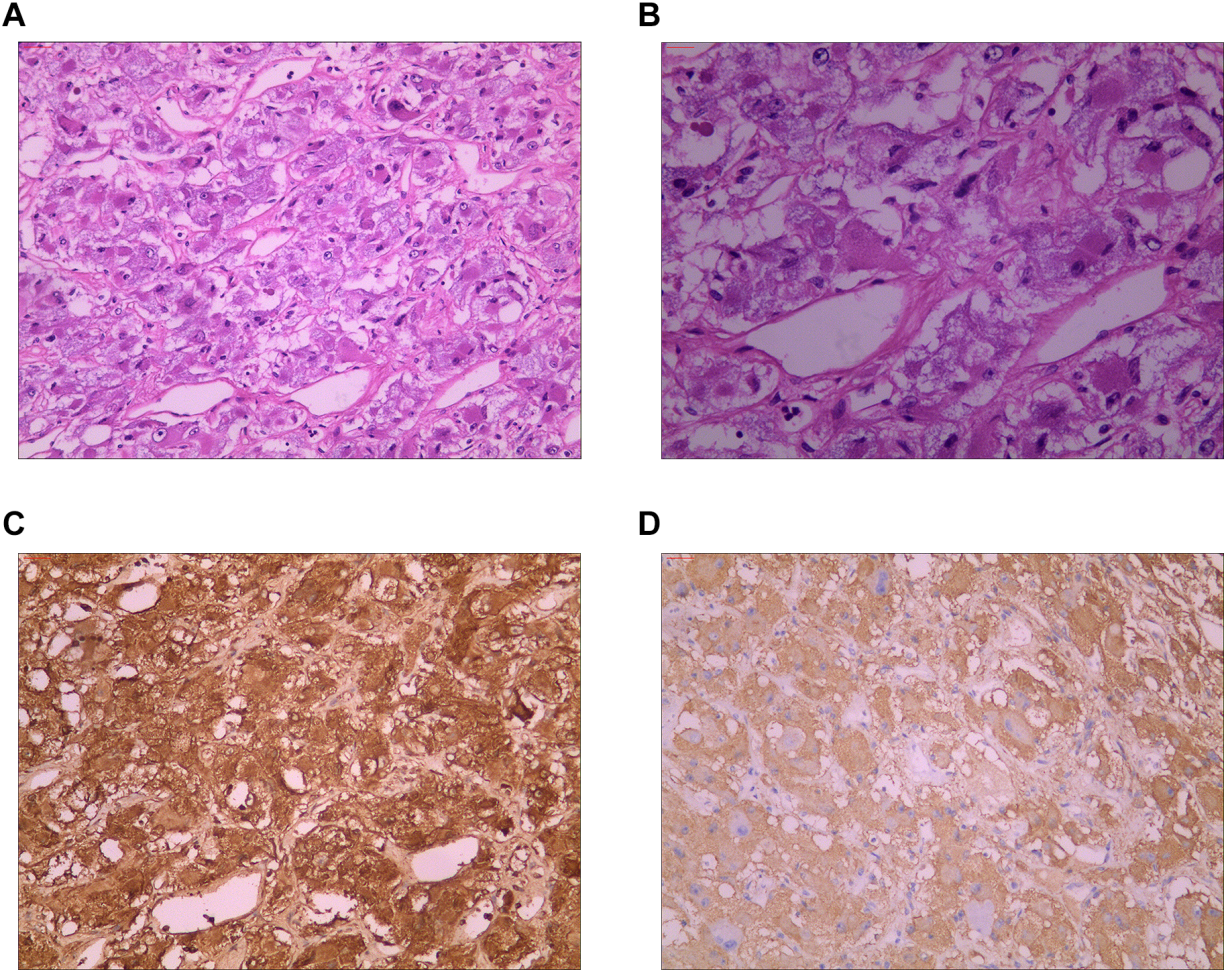
(**A**) Hematoxylin-eosin staining (HE) showing that the tumor cells were distributed in nests and beams, and thin-walled and reticular blood vessels were abundant between the cell nests (10 × 10). (**B**) Hematoxylin-eosin staining (HE) showing that the tumor cells were round or oval at high magnification, finely stained, granular, and evenly distributed, with no mitotic figures observed (10 × 20). (**C**) Immunohistochemical staining showing chromogranin A (CgA) (+). (**D**) Immunohistochemical staining showing synaptophysin (Syn) (+).

After the patient was stable for 14 days, the adrenal mass was treated in the urology department and laparoscopic resection of the adrenal mass was performed. The postoperative tumor pathology report included (left adrenal gland) pheochromocytoma with focal hemorrhage, degeneration, and necrosis (size 6.5 × 5.5 × 3.5 cm). Immunohistochemical staining showed chromogranin A (CgA) (+), S-100 protein (S-100) (+), synaptophysin (Syn) (+), CD56 (+), epithelial membrane antigen (EMA) (−), vimentin (+), GFAP (−), and Ki-67 (+5%) (Figure 4). The patient recovered well after surgery; blood pressure and 24-h urine VMA levels returned to normal after re-examination, and the patient was discharged. The patient tolerated both procedures well. When the pheochromocytoma was removed, the improvement in blood pressure was significant, and the patient eventually stopped all antihypertensive drugs. Six months after surgery, we performed a telephone interview; the patient was in good general condition and had stable blood pressure, which was regularly reviewed at a local hospital, and he participated in light physical work five times a week.

## Discussion

TBAD with pheochromocytoma or adrenal mass is very rare, and there is no relevant literature reporting the prevalence of the combination of the two. Previous studies suggest that pheochromocytomas are rare tumors that, although usually asymptomatic, can be fatal if left untreated (7). The annual incidence rate is approximately 0.6 per 100,000 individuals (2). Pheochromocytomas are tumors in which the adrenal medulla and other chromophobic tissues of the adrenergic system produce excessive amounts of catecholamines. The clinical signs of pheochromocytomas vary; however, the most common are paroxysmal hypertension or paroxysmal exacerbation of persistent hypertension, palpitations, headache, sweating, and other symptoms resulting from catecholamine release.

Aortic dissection secondary to uncontrolled hypertension due to pheochromocytomas is rare. In this case, the pheochromocytoma was discovered incidentally on abdominal ultrasonography and abdominal enhancement CT. The current treatment of choice for pheochromocytomas is surgical resection (8). However, the risk of surgery is greatly increased by cardiovascular accidents due to severe intraoperative blood pressure fluctuations. The role of hypertension in the development of aortic dissection is indisputable as approximately 80% of patients with aortic dissection experience hypertension (9), particularly in patients with highly fluctuating blood pressure.

It is not the entrapment itself that has a fatal effect on patients with aortic dissection, but the series of changes caused by the progression of the dissection, such as pericardial tamponade, aortic rupture and bleeding, and ischemic necrosis of internal organs and extremities. Therefore, the blood pressure of patients with aortic dissection should be strictly controlled. In this case, the patient had both pheochromocytoma and aortic dissection; therefore, we first chose to perform endoluminal repair of the aortic dissection to address the concerns of pheochromocytoma resection. The patient had a functional pheochromocytoma; adequate preoperative preparation can significantly reduce operative mortality (10). Preoperative hypotension, volume expansion, and arrhythmia correction are fundamental measures to reduce perioperative mortality (10). Therefore, we actively administered phentolamine to lower the blood pressure, metoprolol to control the heart rate, and lactated Ringer's solution to expand the volume before surgery.

This is a rare case of pheochromocytoma combined with TBAD. This case suggests that the possibility of pheochromocytoma should be considered in young patients with paroxysmal exacerbations of persistent hypertension. If the patient has a sudden onset of severe tearing pain in the chest and back, the patient should be alerted to aortic dissection, and CTA should be performed urgently to confirm the diagnosis. When young TBAD patients without a previous history of hypertension present with paroxysmal aggravation of persistent hypertension, the possibility of pheochromocytoma complicating TBAD should be considered. Treatment should first address the most life-threatening aortic dissection, followed by pheochromocytoma resection.

## Data Availability

The raw data supporting the conclusions of this article will be made available by the authors, without undue reservation.
