# Supplementary figures and images for: Case report: Pheochromocytoma complicated by type B aortic dissection
Source: Front Cardiovasc Med. 2023 Sep 27;10:1236896. doi: 10.3389/fcvm.2023.1236896 (PMC10565028; doi:10.3389/fcvm.2023.1236896)

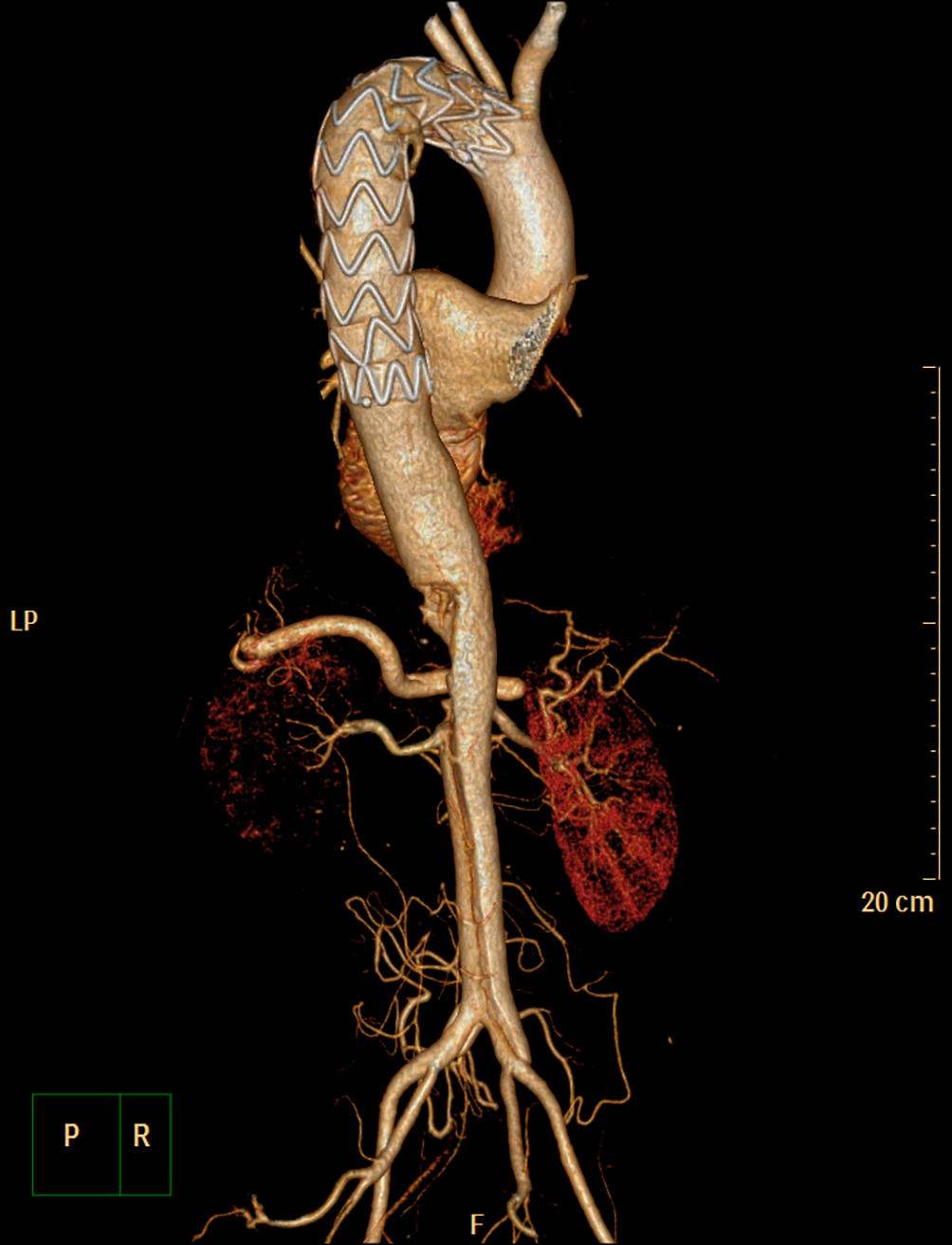

Supplement: Supplementary file 1 [file Image1.tif]
